# Noteworthy Neurological Manifestations Associated With COVID-19 Infection

**DOI:** 10.7759/cureus.14391

**Published:** 2021-04-09

**Authors:** Adam C Kaplan

**Affiliations:** 1 Internal Medicine, St. Francis Medical Center, Trenton, USA

**Keywords:** guillain barre syndrome (gbs), covid 19, bell’s palsy

## Abstract

March 11, 2020 marked the start of the coronavirus disease 2019 (COVID-19) pandemic. COVID-19, caused by severe acute respiratory syndrome coronavirus 2, was being reported as a severe respiratory illness. However, since the recognition of this novel virus, there has been a constant realization that it may present or manifest in a multitude of ways. At first, the typical signs and symptoms were what one would expect from a respiratory virus: cough, shortness of breath, and fever. However, as the disease became more prevalent, neurologic symptoms were reported such as headaches, hypogeusia, and hyposmia. This case report aims to add to the growing body of neurologic manifestations by presenting two cases, Bell’s palsy and Guillain-Barre syndrome. Each case involves flaccid paralysis as the primary presentation.

## Introduction

Severe acute respiratory syndrome coronavirus 2 (SARS-CoV-2) is a newly discovered beta coronavirus that presents with a multitude of different symptoms and signs. The most described presentation is when the virus infects the cells using the angiotensin-converting enzyme 2 (ACE2) receptor [1], leading to respiratory compromise of varying degrees. However, the clinical picture of coronavirus disease 2019 (COVID-19) infection varies widely and SARS-CoV-2 has been implicated in a number of different signs and symptoms that were found to cause widespread systemic infections, of which respiratory complications similar to SARS-CoV-2 were the most recognizable. Nervous system manifestations, including dizziness, headache, hypogeusia, hyposmia, muscle damage, ischemic, and hemorrhage stroke, were also commonly reported [1]. In this report, we present two cases of under-reported neurological complications of the novel coronavirus: Bell’s palsy and Guillain-Barré syndrome (GBS).

Author Notation: Please be advised that this article is a re-publication due to a previous peer review COI that was discovered post publication. This article was formally resubmitted and went through the publication process again in its entirety. It was accepted for publication with no changes or concerns related to the content within the previously retracted article: https://www.cureus.com/articles/35846-noteworthy-neurological-manifestations-associated-with-covid-19-infection/retraction

## Case presentation

Case 1

A 48-year-old female with a past medical history of diabetes mellitus presented with complaints of fever, chills, headaches, fatigue, myalgia, and weakness of one-week duration. She did not have any other contributory past medical, surgical, social, or family history. On physical examination, she was in mild distress secondary to her myalgias. Vital signs showed a temperature of 36.6°C, blood pressure of 175/105 mmHg, pulse of 80 beats/minute, respiratory rate of 18/minute, and pulse oxymetry at 100% on room air. Pulmonary, cardiac, and abdominal examinations were unremarkable on admission. Notably, the neurologic examination was also unremarkable on admission.

**Figure 1:**
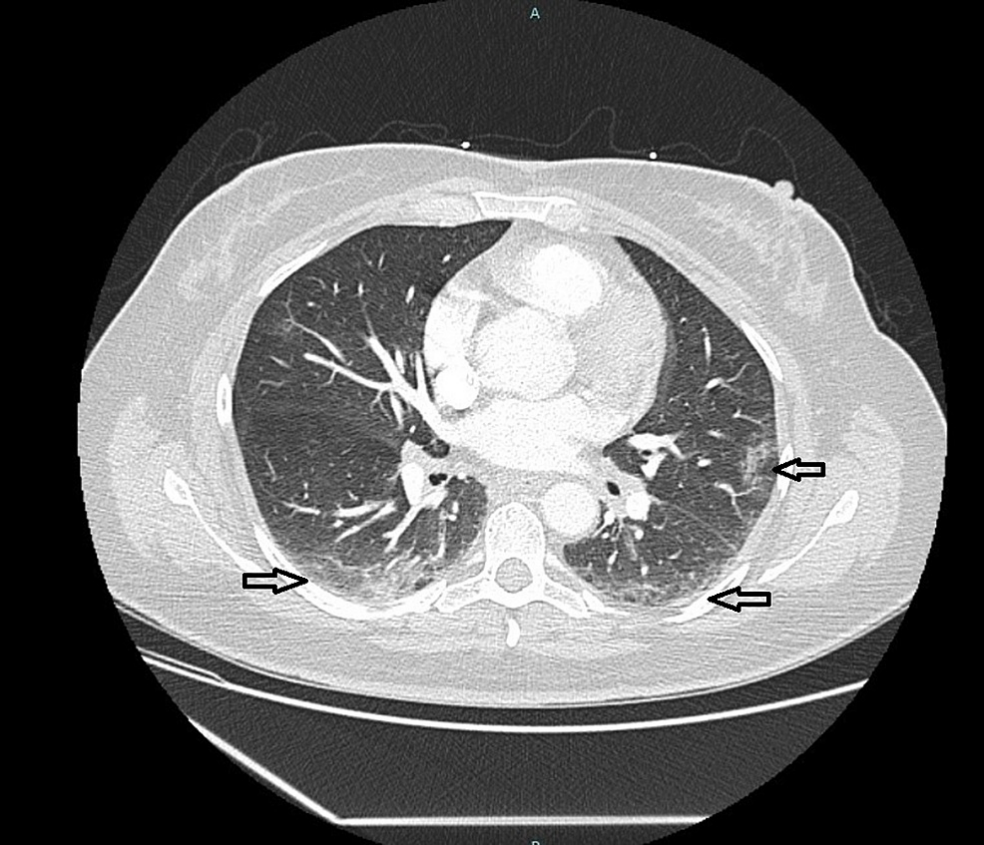
CT scan of the chest with contrast showing bilateral ground-glass opacities consistent with COVID-19 infection. CT, computed tomography; COVID-19, coronavirus disease 2019

The patients’ initial labs revealed a positive SARS-CoV-2 polymerase chain reaction (PCR), sodium of 129 mmol/L (normal range: 135-145 mmol/L), potassium of 3.4 mmol/L (normal range: 3.4-4.7 mmol/L), glucose of 337 mg/dL (normal range: 70-99 mg/dL), and calcium of 9.3 mg/dL (normal range: 8.6-10.2 mg/dL). Other labs showed C-reactive protein of 0.14 mg/dL, D-dimer of 0.67, creatinine kinase of 28, and hemoglobin A1C of 10.9%. The chest X-ray of the patient was unremarkable, but computed tomography (CT) of the chest did reveal ground-glass opacities in the upper and lower bilateral lung fields consistent with a COVID-19 presentation (Figure 1).

The patient was placed on observation status secondary to her SARS-CoV-2 infection. Her hyperglycemia, hyponatremia, and elevated blood pressure resolved on follow-up. On day two, the patient had a complaint of “dry eye” on the left. Examination of the face revealed asymmetric forehead folds, inability to raise the left eyebrow, and left facial droop. There was no bilateral component. The remainder of the neurologic examination was within normal limits. Therefore, a diagnosis of Bell’s palsy was made and the patient was started on empiric steroids (prednisone 20 mg daily for five days), valacyclovir (1 g TID for seven days), and doxycycline (100 mg BID). The doxycycline was discontinued once the Lyme disease titers returned negative and the patient finished her course of empiric valacyclovir and steroids. Upon discharge, there was a marked improvement in her facial asymmetry.

Case 2

A 75-year-old male presented with a two-month duration of progressive worsening of quadriparesis that he noticed after being treated for SARS-CoV-2 infection at another facility. The patient’s past medical history was most notable for a back injury 10 years ago that made him dependent on a wheelchair for most activities, but he was able to ambulate short distances with assistance. In addition, he had hypertension, asthma, and hyperlipidemia that were well controlled. His past surgical, social, and family histories were noncontributory. On physical examination, vitals revealed a temperature of 37.0°C, blood pressure of 128/76 mmHg, pulse of 87 beats/minute, respiratory rate of 22 breaths/minute, and pulse oximetry of 100% on room air. The patient was in no acute distress but his neurological examination was remarkable for the motor weakness of the proximal and distal muscles of all four limbs, which was more pronounced than his historical findings. The weakness was associated with hyporeflexia of the brachial and patellar locations. He had preserved sensation, an equivocal Babinski reflex, and no clonus was elicited. The patient’s cranial nerves, pulmonary, cardiac, and abdominal examination were within normal limits. The patient tested positive for SARS-CoV-2 by reverse transcription-polymerase chain reaction, and the rest of his metabolic panel and complete blood count was unremarkable. The patient underwent CT of the cervical spine, which did not reveal findings that explained the weakness in his bilateral upper and lower extremities. Thus, the patient underwent a lumbar puncture due to suspicion of GBS. The resulting cerebrospinal fluid revealed a normal white count but an albuminocytologic dissociation. This finding, in conjunction with his physical examination, qualified him for level 3, the highest level, on the Brighton criteria of diagnostic certainty for GBS and, therefore, treatment was started.

The patient completed a course of intravenous steroids, 1 g of solumedrol intravenous for five days, and intravenous immunoglobulin (IVIG) at 30 g per day for five days. The patient maintained independent respiratory integrity, and his muscle function improved in concert with physical therapy and treatment. The patient was discharged in a stable condition, with continued physical therapy and outpatient follow-up.

## Discussion

These two cases, GBS and Bell’s palsy, add to the growing number of neurologic sequelae associated with COVID-19, and recognition of their presence may play a role in understanding the respiratory failure associated with SARS-CoV-2 and future therapeutic options.

Bell’s palsy, the final diagnosis of case one, is the most common cranial nerve paralysis, accounting for about two-thirds of all causes of unilateral facial paralysis [2,3]. The typical presentation is well-documented and is distinguished from central causes of paralysis, such as stroke, due to the involvement of the forehead. The exact pathophysiology of Bell’s palsy is still being elucidatedc, but a leading hypothesis is that of an autoimmune nature similar to the pathogenesis of GBS [4,5]. This hypothesis is supported by a paper published in 1975 by Abramsky et al. that showed lymphocytic stimulation by the P1L protein, the same response seen in lymphocytes isolated in patients with GBS [6]. Liston and Kleid further supported this hypothesis by the histologic changes they described: infiltration of the nerve by small round inflammatory cells and breakdown of the myelin sheaths [7].

Our second case was a prototypical presentation of GBS. This entity is an acute immune-mediated polyradiculoneuropathy directed at the peripheral nerves due to molecular mimicry [8]. This process occurs when a foreign antigen, with a similar structure to self antigens, causes the sensitization of the immune system that then directs the response at a similar self-antigen. In the case of GBS, this affects the myelin sheath and nerve conduction becomes impaired. As seen in our patient, the typical manifestations are progressive, ascending, symmetric, flaccid paralysis of the limbs, along with a decrease or absence of deep tendon reflexes [9].

The similarities between these two entities have led some to believe that they are two ends of the same disease spectrum. Despite their similarities, their treatment algorithms are different. Bell’s palsy is focused on decreasing acute inflammation of the facial nerve using steroids [10,11]. Antivirals and antibiotics can augment the steroids if a specific causative agent is suspected such as herpes virus or Lyme disease. Conversely, GBS is treated by either IVIG or plasma exchange therapy to aid in immunosuppression as the proposed mechanism is autoimmune-mediated inflammatory neuritis.

Reviewing our cases revealed a number of questions that may have implications not only for disease recognition but future therapeutics in the fight against COVID-19. We agree with others that Bell’s palsy and GBS are conditions driven by an autoimmune mechanism, molecular mimicry. SARS-CoV-2 may be another virus with similar antigens to our own, leading to molecular mimicry, resulting in these demyelinating neuronal processes. This may explain why there is evidence, albeit of low quality, that the use of IVIG and steroids has a role in the treatment of COVID-19 [12,13], the disease, by immunosuppression and the slowing or stopping of the demyelinating process. Furthermore, COVID-19 does cause a pneumonic process that in and of itself can lead to acute respiratory distress syndrome, for which mechanical ventilation, paralytics, sedatives, and steroids are the mainstay of treatment. These interventions may inhibit our ability to recognize progressive muscle weakness or hyporeflexia that occurs in GBS over the course of days to weeks. The under-recognition could lead to a delay in appropriate treatment and the prolonged ventilator-dependent respiratory courses we are observing in patients with COVID-19 disease.

While GBS and Bell’s palsy are well-recognized entities, there are still many questions that remain unanswered with regards to their pathophysiology and treatment. However, more importantly, the recognition of these entities in an already sick population, such as those with COVID-19, is of utmost importance to ensure the best patient outcomes. We believe that further research should be performed to clarify the association, correlation, or causation of COVID-19 and demyelinating neuropathies; establish the prevalence of these demyelinating neuropathies in the COVID-19 patient population; and be aware that these conditions, particularly GBS, can occur in conjunction with direct lung injury and may play a role in refractory respiratory failure observed in patients with COVID-19.

## Conclusions

COVID-19 infection can present with different neurological manifestations; Bell’s palsy and GBS are becoming more recognized worldwide. A detailed neurological examination on initial presentation and on follow-up is crucial to identify any potential neurological condition as timely diagnosis and prompt treatment will prevent the consequences of these progressive neurological diseases, some of which are life-threatening.

## References

[1] Zhao Y, Zhao Z, Wang Y, Zhou Y, Ma Y, Zuo W (2020). Single-cell RNA expression profiling of ACE2, the receptor of SARS-CoV-2. Am J Respir Crit Care Med.

[2] Peitersen E (2002). Bell’s palsy: the spontaneous course of 2500 peripheral facial nerve palsies of different etiologies. Acta Otolaryngol Suppl.

[3] Greco A, Gallo A, Fusconi M, Marinelli C, Macri GF, de Vincentiis M (2012). Bell's palsy and autoimmunity. Autoimmun Rev.

[4] Charous DI, Saxe BI (1962). The Landry-Guillain-Barre syndrome. Report of an unusual case, with a comment on Bell's palsy. N Engl J Med.

[5] Abramsky O, Webb C, Teitelbaum D, Arnon R (1975). Cell-mediated immunity to neural antigens in idiopathic polyneuritis and myeloradiculitis. Clinical-immunologic classification of several autoimmune demyelinating disorders. Neurology.

[6] Abramsky O, Webb C, Teitelbaum D, Arnon R (1975). Cellular immune response to peripheral nerve basic protein in idiopathic facial paralysis (Bell’s palsy). J Neurol Sci.

[7] Liston SL, Kleid MS (1989). Histopathology of Bell's palsy. Laryngoscope.

[8] Yuki N, Tagawa Y, Handa S (1996). Autoantibodies to peripheral nerve glycosphingolipids SPG, SLPG, and SGPG in Guillain-Barré syndrome and chronic inflammatory demyelinating polyneuropathy. J Neuroimmunol.

[9] Sejvar JJ, Baughman AL, Wise M, Morgan OW (2011). Population incidence of Guillain-Barré syndrome: a systematic review and meta-analysis. Neuroepidemiology.

[10] Grogan PM, Gronseth GS (2001). Practice parameter: Steroids, acyclovir, and surgery for Bell's palsy (an evidence-based review): report of the Quality Standards Subcommittee of the American Academy of Neurology. Neurology.

[11] Sullivan FM, Swan IR, Donnan PT (2007). Early treatment with prednisolone or acyclovir in Bell's palsy. N Engl J Med.

[12] Galeotti C, Kaveri SV, Bayry J (2017). IVIG-mediated effector functions in autoimmune and inflammatory diseases. Int Immunol.

[13] De Ranieri D, Fenny NS (2017). Intravenous immunoglobulin in the treatment of primary immunodeficiency diseases. Pediatr Ann.

